# Developing a theoretical framework for enhancing green project approaches via Agile methodology

**DOI:** 10.1038/s41598-024-78613-x

**Published:** 2024-11-13

**Authors:** Ahmed. Ali, Hesham. Sameh

**Affiliations:** Faculty of Engineering, Cairo University, Giza, Egypt

**Keywords:** Green projects, BIM, GBIM - AGILE, 6 SIGMA, SCRUM, Civil engineering, Environmental sciences, Environmental social sciences

## Abstract

The application of green building approaches has significantly increased in many countries and buildings, accompanied by the development of numerous standards and methodologies for their implementation. Despite these efforts, not all risks and challenges facing green projects have been eliminated in some regions. Issues such as financing difficulties, budget overruns, and schedule delays continue to pose significant challenges. As a result, no substantial improvements have been made in the approach to green projects. Therefore, this study aims to develop a theoretical framework that enhances the approach to green projects. By utilizing the AGILE methodology, specifically SCRUM, the most significant risks facing green projects were identified and converted into indicators based on prior studies. Furthermore, Building Information Modeling (BIM) technology, particularly Green BIM (GBIM), was employed to create tools and elements to address these challenges, leading to the development of a conceptual framework capable of providing solutions. This framework was built around 30 indicators, whose weights were determined through an electronic questionnaire involving 140 specialists in green projects, AGILE methodology, and BIM technology. Assistance from academic experts was also sought. The results of the questionnaire, analyzed using statistical methods, contributed to the development of a theoretical framework aimed at improving the approach to green projects.

**Supplementary Information:**

The online version contains supplementary material available at 10.1038/s41598-024-78613-x.

## Introduction

In order to attain sustainability, green construction necessitates approaching structures from an environmental, social, and economic standpoint^1^. It is used starting at the design phase and continuing through the implementation, operation, and waste disposal stages^2,3^. Green projects currently face a number of risks and problems during implementation and operation^4,5^, such as project delays caused by unforeseen obstacles resulting from poor project management, project costs that exceed initial estimates, inadequate project financing, incomplete knowledge of the materials to be used, inadequate team selection ranging from the first project manager to the worker, and, finally, a lack of interest from some countries in the type of these significant projects^6^. Numerous studies conducted in China, Uzbekistan, and Singapore have clarified these issues; however, each study’s problem was addressed independently without addressing and projecting future problems^7,8^. This is where the AGILE methodology comes into play, as it can effectively predict and solve all problems through continuous improvement^9^. It is challenging to apply to these kinds of projects without the use of a tool like BIM, particularly the GBIM related to green projects, because it can accurately provide the information needed for the project, classify and organize it, present the issue in an understandable manner, and offer tools to address it. Here, we require a straightforward, well-structured, and dynamically updated conceptual framework that can classify and arrange BIM for data and information derived from green projects, thereby anticipating and comprehending all the issues that arise in green projects. This framework should be built on the Agile methodology and fundamentals.

The aim of this study is to provide a theoretical framework that can anticipate and address every problem that could emerge in the course of green initiatives. This will address every issue, streamline the management of these projects, and improve sustainability from the planning through to the execution phases. We will employ the SCRUM strategy, which is most suited for our research, together with the AGILE technique. This approach is effective in creating a framework that the GBIM system can utilize to produce indications that are useful for managing and resolving issues that crop up in green projects. Additionally, it will be updated often, which will aid in resolving any issues that may come up in the future. First, we will look at green projects and determine the most important success elements for them in this study. We will then examine the main hazards they face using data from earlier research projects carried out in different nations. Our aim is to gain a thorough understanding of the difficulties faced by green initiatives through this method, which will subsequently be converted into quantifiable indicators (30 in total). By utilizing the GBIM system and the AGILE framework’s SCRUM methodology, we will build a theoretical framework that is intended to successfully handle these difficulties. To confirm this, an electronic questionnaire will be administered, involving experts and scholars in the domain who will assess the markers.

## Literature review

### Introduction

Sustainable development has become a priority in numerous countries, driving the adoption of green projects that aim to reduce environmental impact and enhance energy efficiency^8–10^. Simultaneously, the use of Agile project management has risen across industries, valued for its adaptability and focus on collaborative work^11^. Green Building Information Modeling (GBIM) is a crucial technology in modern construction, streamlining the implementation of sustainability principles in project design and execution^12^. However, there is a notable lack of research that integrates all three elements—green projects, Agile management, and GBIM—into a cohesive framework. This literature review critically assesses current studies, highlights research gaps, and explores how the combination of these approaches could enhance the management and efficiency of green projects.

### Green projects

Green projects focus on minimizing environmental impact through sustainable design, construction, and operation practices. They aim to reduce resource consumption and emissions while promoting the use of renewable energy, smart materials, and environmentally friendly techniques^13,14^.

#### Current challenges in green projects

Research has highlighted several challenges in green project implementation. For instance, studies like those focusing on green projects in Singapore and Uzbekistan have identified economic, administrative, and technical barriers^6,7^. Green projects often face cost overruns, time management issues, and knowledge gaps among project participants^15,16^. In particular, a lack of understanding of green building concepts by key stakeholders, including engineers and workers, poses a significant challenge^16,17^. Additionally, limitations in legal frameworks and interdepartmental cooperation have hindered green project scalability, especially in countries like Uzbekistan^7,18^.

### Agile project management

**Fig. 1:**
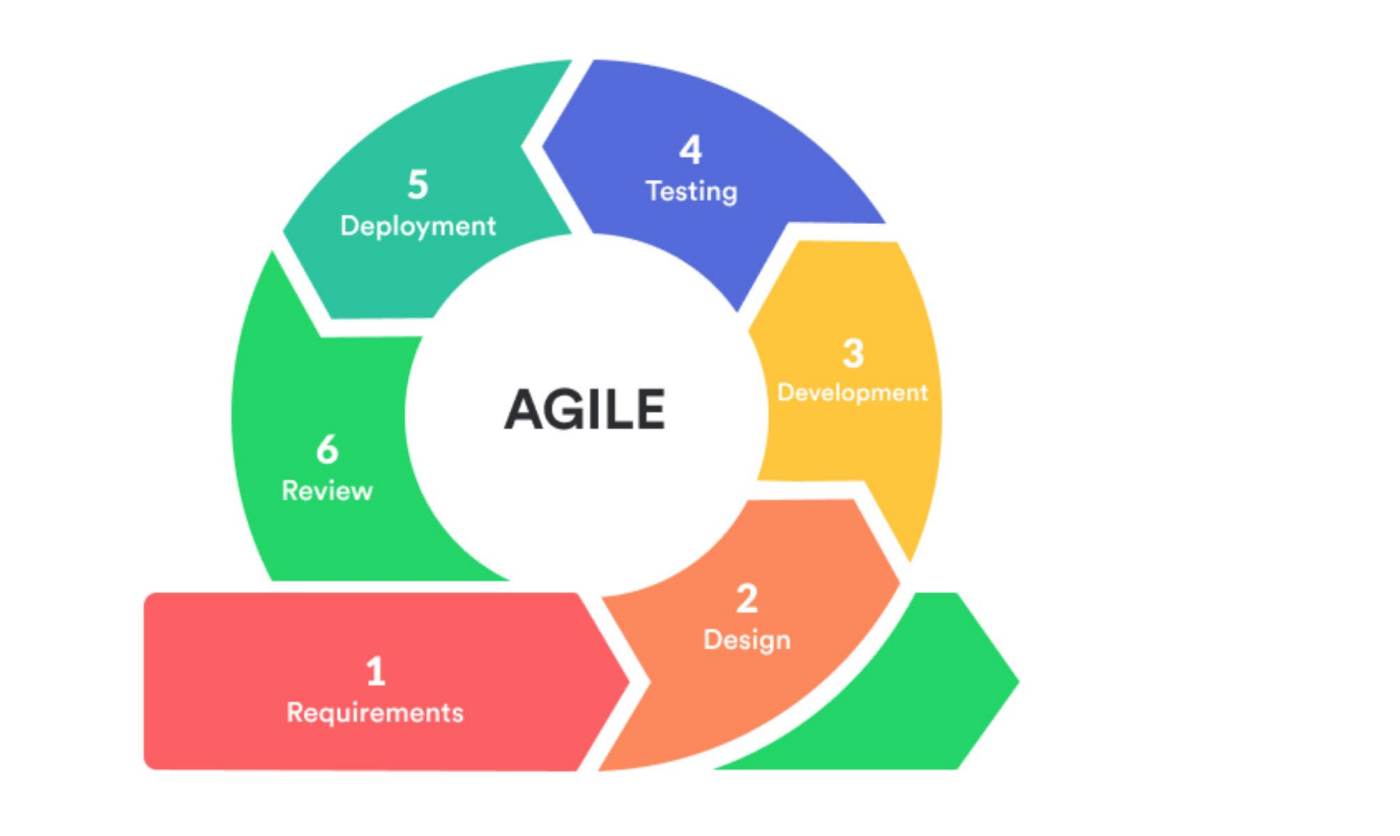
Illustrates the Agile methodology, starting from requirements until reaching the review, and it can be repeated many times by https://www.krasamo.com/agile-development-process/accessed in 04/03/2024.

Agile project management, originally developed for the software industry, emphasizes flexibility, collaboration, and iterative development^19,20^. It has been adapted for other fields, including construction, where it has shown promise in improving project management efficiency^9,21,22^. Agile follows a methodology that can be repeated multiple times until the task is completed successfully and the aim is achieved, beginning with defining requirements and continuing through the review process^20,23^, as illustrated in Fig. 1.

#### Current challenges in green projects

Agile methodologies, such as Scrum, have been applied to green construction projects to enhance flexibility and performance^11,24^. Scrum facilitates rapid iterations and continuous improvements, which are particularly beneficial in green projects where sustainability standards and technological advancements require frequent updates^25^. However, research indicates that the application of Agile in construction is still limited, with many studies calling for a deeper exploration of its benefits in project design and implementation^21^.

#### Workflow of scrum

**Fig. 2:**
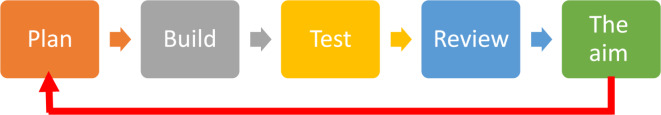
Shows the Scrum model’s work cycle, starting from the plan until achieving the aim and then repeating it again by Srivastava, A., Bhardwaj, S., & Saraswat, S. (2017, May). SCRUM model for agile methodology. In 2017 International Conference on Computing, Communication and Automation (ICCCA) (pp. 864–869). IEEE.

Scrum is a flexible and lightweight framework that combines both iterative and incremental approaches, enabling continuous improvement in green projects while mitigating problems and risks through rapid development^26,27^. It allows teams to adapt to ongoing changes while maintaining a focus on performance, quality, and strong collaboration with clients and team members. Scrum follows an iterative process, beginning with planning and continuing through goal achievement, repeating the cycle until the process reaches its maximum potential for success^28^, as illustrated in Fig. 2.

#### Agile methodologies in the construction

Agile methodologies, originally developed for software development, have been increasingly applied in the construction industry, particularly to address the complex and unpredictable nature of construction projects. The adaptability and iterative nature of Agile frameworks like Scrum offer valuable tools for managing construction projects where project requirements often evolve over time. Research demonstrates that the use of Agile practices in construction improves flexibility and communication among project stakeholders, facilitating real-time decision-making and adjustments based on feedback from clients and project teams^29,30^. Agile has shown to be particularly effective in reducing delays and cost overruns, as it allows for incremental project delivery and more accurate forecasting^31,32^.

In recent years, the integration of Agile methodologies with sustainability efforts in construction has become a significant area of research. Studies indicate that Agile frameworks, when combined with green building practices such as Green Building Information Modeling (GBIM), can significantly improve sustainability outcomes. The iterative feedback loops in Agile allow for continuous refinement of sustainability measures throughout the project lifecycle. For example, regular project reviews in Scrum enable teams to adjust plans for energy efficiency, waste reduction, and resource optimization^29,33^. This synergy between Agile and sustainable construction has been particularly noted in residential projects, where environmental regulations and consumer demand for sustainable buildings are on the rise^33,34^.

Despite these advantages, Agile application in construction is not without challenges. The traditional linear and hierarchical structure of construction management often conflicts with Agile decentralized and flexible approach^30^. A major barrier to Agile adoption in construction is the lack of familiarity among industry professionals. Additionally, large-scale construction projects may struggle to fully integrate Agile practices, particularly in the context of workflow automation and coordination across multiple teams^31^. However, as awareness of Agile benefits grows, there is a movement toward developing hybrid project management models that combine Agile principles with more traditional construction methodologies^35^.

##### Limitations of Agile in construction

Despite its potential, Agile methods face challenges in large-scale construction projects. the complexity of integrating Agile with traditional construction management systems, as well as issues related to workflow automation, have been highlighted in studies such as those conducted in Singapore and Washington D.C^36^. Additionally, Agile methods like Scrum are often underutilized due to a lack of research on their scalability across entire project lifecycles^28^.

### Green Building Information Modeling (GBIM)

GBIM integrates sustainable principles with Building Information Modeling (BIM) technology, creating a powerful tool for designing and managing green projects^37,38^. BIM’s multidimensional modeling capabilities support various stages of the construction process, from conceptual design to lifecycle maintenance^39,40^.

#### GBIM in green projects

GBIM has been praised for its ability to enhance project efficiency and sustainability. Research has shown that GBIM can reduce energy consumption, improve resource management, and streamline the green certification process^12,41^. However, its application in construction remains limited, particularly in developing countries. Studies have pointed out challenges in integrating all green building aspects into BIM systems, with a lack of standardized strategies and methodologies for effective implementation^42^.

#### Dimensions of BIM

**Fig. 3:**
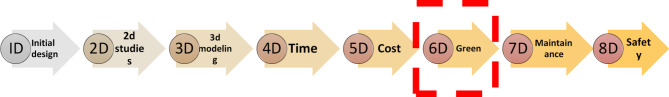
8 dimensions for BIM, each dimension has its function indicated, especially the sixth dimension and its function, the green architecture by https://www.virtualbuildingstudio.com/bim-dimensions-2d-3d-4d-5d-6d-7d-8d/ accessed in 17/05/2024.

BIM technology is categorized into eight dimensions that cover the entire project lifecycle, from the concept stage through to maintenance, operations, and safety^43^. Among these dimensions, the sixth dimension stands out as the most significant because it represents the green approach, often referred to as GBIM^44^, as illustrated in Fig. 3. This classification was primarily used in the theoretical framework.

### Research gap

Despite the advances in green projects, Agile management, and GBIM, there is a significant gap in the literature concerning their integration. Few studies have explored how these three elements could be combined to improve project outcomes. Most research focuses on these areas in isolation, limiting their potential impact on construction sustainability. This research aims to address this gap by developing a theoretical framework that combines Agile, green projects, and GBIM to enhance project management and execution.

### Critical analysis of current studies

While existing research offers valuable insights into green project management, Agile methodologies, and GBIM, it also reveals significant limitations. Many studies are geographically limited, focusing on specific countries like Singapore or Uzbekistan, which reduces the generalizability of their findings^6,7^. Additionally, the adoption of Agile methodologies in construction has been slow due to a lack of research on its long-term scalability^21^. GBIM, though promising, faces challenges in implementation due to technical complexity and the absence of industry-wide standards^12^. The top 30 risks associated with green projects are summarized in the literature, as detailed in Table 1.

Furthermore, a key limitation across many studies is the lack of comprehensive strategies for integrating sustainability into construction management. While Agile and GBIM offer potential solutions, their application to green projects has been underexplored. This research proposes a novel approach that integrates these three elements, addressing the gaps in current literature and providing a robust framework for sustainable construction.

In conclusion, Table 2 in the research provides a comprehensive summary of all the studies discussed in the literature review. Each reference is summarized based on the key limitations, objectives, research gaps, and methodologies, along with a summary overview of each study.

**Table 1:** 30 risks by author.

	Stakeholders	No.	Risks
**1**	**Project manager**	1	Inaccurate studies^6^
		2	Unpredictability^18^
		3	High cost of green projects^45^
		4	Lack of knowledge of green thought^2^
		5	Lack of interest in the life cycle of the building^2^
		6	Safety and accidents^6^
**2**	**Contractor**	1	Delay in project delivery^46^
		2	Lots of waste during implementation^8^
		3	Lack of experience and quality^45^
		4	Poor site management^6^
**3**	**Labor**	1	Labor productivity^6^
		2	Hazards facing workers on site^16^
**4**	**Designer**	1	Poor team experience^1^
		2	Poor in design^6^
		3	Errors in deign^7^
		4	Clashes in the design^16^
		5	Delay in project delivery^47^
		6	Lack of knowledge of materials related to thermal and acoustic comfort^15^
		7	Not knowing the materials that extend the life of the building^18^
**5**	**Owner**	1	Non-cooperation^48^
		2	Continuous change^16^
		3	Hesitation in making decisions^6^
		4	Lack of a clear financing strategy^7^
		5	Lack of knowledge of the value of green projects^48^
		6	Failure to set flexible programs to reduce waste^47^
**6**	**Consultants**	1	Errors in the pricing process^18^
		2	Error in the review process^48^
**7**	**Governmental ** **institution**	1	Change in regulations^6^
		2	Lack of awareness of the importance of green projects^8^
		3	Lack of interest in feasibility studies^15^

**Table 2:** Comprehensive summary of literature review research by author.

Title	Limitations	Objectives	Gap	Method	Summary
Risk assessment: A case of Singapore’s green projects	Low response rate to the survey, limited geographical scope (Singapore)	Assess risks in Singapore’s green building projects using a fuzzy synthetic evaluation (FSE) model	Limited studies on risk management in green building projects	Questionnaire survey from project managers, FSE risk assessment model	Risk management study that identifies the most critical risks in green building projects in Singapore.
Uzbekistan’s Transition to a Green Economy, Problems, and Solutions	Challenges with effective policy implementation, legal gaps, and interdepartmental cooperation	Analyze problems and provide solutions for Uzbekistan’s green economy transition	Lack of comprehensive strategies and cooperation in implementing green policies	Analytical review of existing policies, legal frameworks, and ecological programs	Focuses on Uzbekistan’s transition to a green economy, discussing the importance of sustainable practices and policies.
The present situation, problems, and countermeasures of green investment in China	Financing channels are limited and costly; low leverage of financial funds	Understand the current green investment situation and provide countermeasures	Insufficient financing channels and inefficiency in the green investment mechanism	Statistical analysis of green investments and related activities, covering six main investor groups	Discusses green investment in China and proposes solutions for expanding financing channels and improving efficiency.
Green building standards and Certification systems	Time and resource constraints for data collection and modeling, subjectivity in impact result conversions.	Enhance green building standards, improve energy and environmental performance in the construction sector.	Limited data availability, especially for new technology like nanotech, lack of unified green product definitions.	Life Cycle Assessment (LCA) to evaluate environmental burdens across a product’s lifecycle.	Focuses on enhancing building sustainability by adopting green building standards and certifications to reduce carbon emissions and energy consumption.
Green Project Management	Gathering reliable inventory data is resource-intensive; subjective decisions in LCA scoring.	Guide project managers in incorporating sustainability into project decisions.	Insufficient transparency in LCA data modeling and varied methodologies.	Use of frameworks like ISO 14,040 and software tools like SimaPro and GaBi for conducting LCA.	Discusses the integration of green techniques in project management, particularly through lifecycle assessments to identify high-impact areas and improve sustainability in projects.
Green projects: An information drives analysis of four cases	Lack of environmental policy, insufficient managerial support for green efforts.	Enhance efficiency in project management by focusing on sustainability and environmental risk management.	Difficulty in securing management commitment and funding for green initiatives.	Use of decision-making tools like SMARTER and greenality principles to guide sustainable project execution.	This document focuses on how to run projects efficiently while integrating sustainability measures, emphasizing a framework for implementing and monitoring green initiatives in project management.
Engaging Consultants in Green Projects in Malaysia	Data limited to Vietnam and developing countries; results are subjective due to participant judgment	To develop a risk assessment (RA) model for Green Building (GB) projects in Vietnam	Lack of research on risk assessments for GB projects in developing countries	Developed an RA model based on impact level, probability, and RM, applied to GB projects in Vietnam	This paper develops a practical RA model to assess GB project risks in Vietnam, identifying critical risk factors and proposing mitigation strategies
Managing Green Building Development: Singapore Case Study	Project was constrained by legal and environmental regulations	Explore legal and environmental challenges in renewable energy projects like the Open View Solar Farm	Lack of clear integration between environmental and development policies	Analysis of legal and policy documents on environmental regulations for solar projects	This document examines the challenges and legal obstacles faced by solar farm projects within the U.S. regulatory framework
Risk Assessment Model for Green Building Projects in Vietnam	Limited credible research on benefits of green buildings; high cost of green practices	Compare conventional and green building processes; Identify challenges in green building construction	Need for more research on green building benefits and communication among project members	Literature review and interviews with project managers to identify common challenges and solutions	The paper identifies cost, communication, and research challenges in green projects and proposes solutions like government incentives and regular meetings
Sustainable Construction: Green vs. Conventional	Based on Malaysian context, limiting generalization; lack of green consultants	Explore engagement methods for project consultants in Malaysian green projects	Limited research on how consultants are engaged in green construction	Qualitative interviews with 18 respondents from green-certified projects	This paper examines consultant engagement methods in green projects, comparing client-driven and architect-driven approaches, and highlighting selection criteria
The Effectiveness of Green Building Initiatives	The extra work in green projects introduces complexity in planning and managing.	To assess the additional tasks and risks involved in green building projects in various stages.	There is limited understanding of the extra work required in green building projects.	Case study approach analyzing tasks across different green building development stages.	Explores the additional tasks required in green building projects, focusing on legal, market, and technical complexities during project implementation
Green Building Development Process: Challenges and Opportunities	The limited expertise in green building practices poses a challenge.	To identify key challenges and opportunities in green building development.	A lack of experienced consultants and professionals in green building projects.	Qualitative interviews with project managers, architects, and contractors involved in green building development.	This study focuses on the knowledge gaps and challenges faced in the green building sector, emphasizing the need for better expertise and consultant engagement strategies.
Agile Manifesto	Lack of documentation isn’t eliminated but reduced; Agile is context-specific, not a universal solution	Promote agile methodologies focusing on collaboration, adaptability, and working software	Resistance to agile due to misconceptions of it being “light” or a “hacker” approach	Creation of Agile Manifesto and principles, formed by 17 experts through discussions and practice	The Agile Manifesto emphasizes collaboration, responding to change, and working software over rigid documentation and processes.
Using Six Sigma to Improve Outcomes in Higher Education	Limited findings on the long-term impact of Six Sigma in educational environments	Implement Six Sigma methodology in higher education to improve student outcomes	The application of Six Sigma in education is under-explored and lacks cost/benefit analysis	DMAIC methodology (Define, Measure, Analyze, Improve, Control) applied in two phases in an educational setting	Six Sigma was applied in an educational institute to improve processes, showing potential for enhancing outcomes, though further analysis is required.
Green Building Project Management: Obstacles and Solutions for Sustainable Development	High initial costs, lack of green product info, complex legislation, and unequal benefits distribution	Identify obstacles in green building project management and propose solutions to encourage sustainable construction	Lack of a proper project management framework for green building projects in Singapore	Surveys and interviews with 31 experts; review of existing frameworks and project delivery systems	Examines challenges in managing green building projects and proposes solutions like widening government incentives and developing a PM framework.
Agile Six Sigma in Healthcare: Case Study at Santobono Pediatric Hospital	Focus on a single hospital unit; tested only on infant neuropsychiatry, with no classical statistical pre–post analyses	Improve patient access to clinical services using Agile and Six Sigma approaches	ack of efficient models to handle patient no-shows	Discrete event simulation (DES) with DMAIC methodology	Combines Agile and Six Sigma to improve patient absenteeism management, applying a simulation model to test overbooking strategies
An Analytical Approach in Usage of Agile Methodologies in Construction	The new method brings confusion and ambiguity when implemented in traditional setting	Develop a controlled framework to handle the dynamic nature of construction projects	Poor application of agile methods in construction; delays due to design & change orders	Application of Agile management (Scrum, sprints) in construction	Agile methods are applied to construction management to better handle change orders and improve project outcomes
Comparative Analysis of Project Management Approaches	Comparison only between existing frameworks, with limited practical implementation	Analyze and compare Agile, Lean, Six Sigma, and Traditional PM methodologies	Existing literature lacks a comprehensive comparison of all four approach	Literature review and comparative analysis​	A detailed comparison of Agile, Lean, Six Sigma, and Traditional PM to find strengths and areas for integration
Systematic Review of Agile Methodology	Sampling process may limit the robustness of findings	Bridge the gap between practical and theoretical interpretations of Agile	No prior systematic literature review with a temporal perspective	Systematic literature review with text mining and contingency analysis	Reviews the evolution of Agile and highlights under-researched areas in management theories
Agile Project Management for Design-Build Construction Projects: A Case Study	Waterfall Project Management is still dominant despite DB methods. Scrum limited by a lack of research on its application throughout entire DB lifecycle.	To explore Scrum’s applicability to Design-Build projects, aiming to enhance communication, cost, and schedule control.	Lack of research on applying Agile (Scrum) to Design-Build projects, particularly during the entire design and construction phases.	Case study applying Scrum to a Design-Build school project in Washington D.C.	Demonstrates Scrum’s potential benefits in cost and schedule control for Design-Build projects but calls for further research into scalability across whole project lifecycles.
Innovation by Integration of Drum-Buffer-Rope (DBR) Method with Scrum-Kanban	Kanban cannot predict bottlenecks in the flow, leading to potential workflow stoppage.	To integrate DBR with Scrum-Kanban to improve throughput in Agile Project Management.	Lack of research integrating DBR with Scrum-Kanban in the context of agile project management.	Monte Carlo simulation of Scrum-Kanban combined with DBR to test four workflow scenarios.	Shows that integrating DBR with Scrum-Kanban increases throughput and minimizes workflow bottlenecks, with optimal performance in the modified “Unbalanced Line” scenario.
Agile Methods Comparison: Kanban vs. Scrum vs. XP	Scrum struggles with large-scale automation, workflow, and testing, especially in complex or distributed projects.	To compare Scrum, Kanban, and XP in terms of applicability to various types of projects.	Difficulty in scaling Agile methods for larger, complex, and distributed projects.	Comparative analysis based on the application of Scrum, Kanban, and XP in different types of agile environments.	Each method has distinct strengths: Scrum excels in managing cross-functional teams, Kanban in workflow visualization, and XP in rapid iterative feedback for software.
Scrum Model Scaling for Large Distributed Projects	Lack of automation and experienced team members in Scrum leads to inefficiencies in large projects.	Propose a scalable Scrum model for large, distributed projects.	Challenges in scaling Scrum to larger, distributed teams, particularly in testing and workflow automation.	Modified Scrum model addressing workflow and team structure issues in large, distributed projects.	Proposes scaling solutions for Scrum in large projects by separating traditional and innovative tasks, though challenges remain in automation and team management.
Agile Methodology in Education of IT Students	Lack of precise Agile definitions, especially in non-IT projects	Teach IT students Agile methodology through real-world projects and Scrum applications	Inconsistent definitions of Agile	Real-world application of Agile and Scrum in education	Discusses the application of Agile methodologies like Scrum and Kanban in IT education, focusing on teamwork, incremental development, and iterative approaches
Scrum Project Management	Difficulties with integration of conventional and Scrum methods	To implement Scrum and Agile for effective project management in various industries	Need for a balance between traditional and Agile methodologies in management	Scrum framework implementation, sprints, and daily Scrum meetings	Highlights the use of Scrum in project management to improve efficiency and adaptability
Developing Agile Skills in IT Courses	Limited to IT-specific applications, may not translate well to non-IT disciplines	Teach Agile methods like Scrum to IT students	Lack of Agile training at non-IT departments	Interactive workshops, real-world Scrum team simulations	Describes teaching methods aimed at imparting Agile development skills through simulations, coding exercises, and iterative teamwork
Challenges and Success Factors for Large-Scale Agile Transformations	Large-scale transformations face coordination difficulties between teams	Analyze success factors for Agile transformations in large organizations	Limited studies on large-scale Agile applications​	Systematic literature review of large-scale Agile transformations	A review of challenges and key success factors for scaling Agile in larger organizations, focusing on coordination, communication, and resource management​
BIM in Principle and in Practice” (Barnes & Davies)	BIM adoption is slow due to technical complexity and lack of industry-wide standards.	To explain the basic principles of BIM and its practical application in various construction projects.	Limited understanding of BIM among many in the construction industry.	Review of BIM principles, case studies of BIM applications in railway stations and precast concrete projects.	Provides a comprehensive introduction to BIM, discussing its potential to revolutionize the construction industry through improved collaboration and efficiency. Highlights case studies of BIM applied to complex projects.
Green BIM: Future Potential for Green Building Projects	Difficulty in integrating all green building aspects into BIM; limited use in construction due to lack of standardized strategies.	To explore how BIM can be applied to green building projects to improve sustainability and streamline certification processes.	Gap between academic research on BIM and practical use in green building construction.	Literature review, analysis of Green BIM applications in building design, and potential for integration with certification systems like LEED.	Examines how BIM can be used to support green building projects, improve energy efficiency, and streamline certification. It emphasizes the potential for using BIM in life-cycle assessment (LCA) and sustainability measures for future construction.
The Role of BIM Technologies in Education Systems	Challenges in integrating BIM into educational environments due to varying levels of technological infrastructure in institutions.	To highlight the importance of BIM for developing technological competence in engineering and education.	Lack of educational consortia and global educational resources using BIM.	Review of educational processes and the integration of BIM technologies in curricula for engineers and educators.	Discusses the necessity of incorporating BIM technologies in the educational system to foster the technological competence required for future engineers. It promotes BIM’s role in practical training and addresses the creation of an open information-educational environment for collaboration and global knowledge-sharing.
BIM Benefits and its Influence on BIM Implementation in Malaysia	Lack of awareness and proper understanding of BIM benefits among construction companies	To identify significant BIM benefits influencing implementation and scrutinize the relationship between BIM benefits and implementation in Malaysia	Lack of comprehensive research evaluating Malaysian construction stakeholders’ perception of BIM benefits	Survey of construction professionals in Malaysia (questionnaires)	Explores BIM’s potential to increase productivity, time, cost savings, and enhance communication among stakeholders
Employ 6D-BIM Model Features for Building Sustainability Assessment	Economic, technical, and awareness-related constraints in integrating BIM with sustainability in Iraqi construction projects	To investigate the benefits of BIM as a project management tool for building sustainability	Lack of BIM integration with sustainability in Iraq	Framework based on ISO 1440, detailing phases from 3D to 6D	Explains the 6D BIM model’s potential for sustainability, including lifecycle costing and energy performance analysis
Integration of BIM Dimensions for Project Management and Sustainability	High initial costs, insufficient industrial development, and lack of trained BIM engineers	To explore BIM’s dimensions from 1D to 10D and their impact on construction efficiency and sustainability	Insufficient adoption of BIM’s higher dimensions (above 5D)	Review of literature on BIM dimensions and their applications	Analyzes BIM’s extended dimensions, including industrialized construction (10D), sustainability (6D), and building management (7D)
Research on Collaborative Design System of Green Building Information Model	Limited collaboration between designers, weak information flow in traditional design systems.	To establish a collaborative green building information model (GBIM) decision-making system.	Traditional architectural design hinders green building collaborative efforts, affecting efficiency and sustainability.	IDEF0 method, traffic simulation, building information modeling (BIM)	Proposes a GBIM-based decision-making system to improve resource utilization and coordination throughout a building’s lifecycle.
Information Flow Modeling Technology of GBIM	Complex and chaotic information flow in green building projects, difficulty in handling information exchanges efficiently.	To introduce GBIM with information flow technology for improved green building design and management.	Lack of a systematic and efficient method to manage information flow and task coordination in green building projects.	IDEF0v modeling technique, hierarchical graph structure	Explores using information flow modeling to improve task coordination, ensuring efficiency in green building designs through data management and task synergy.
The Level of Importance of Criteria and Sub-Criteria in Green Building Index Malaysia	Limited local adaptability of green building evaluation systems, disparity between current criteria and actual needs.	To evaluate the importance of different criteria and sub-criteria in the Green Building Index Malaysia (GBIM).	Current GBIM places undue emphasis on energy efficiency, while other criteria, like indoor environmental quality, are undervalued.	Analytical Hierarchy Process (AHP), pair-wise comparison	Assesses the importance of criteria like energy efficiency and indoor environmental quality in Malaysia’s GBIM system, suggesting recalibration of priorities.
Feasibility Study of Agile in Construction	Limited flexibility in adoption, lack of empirical evidence for construction	Assess Agile’s benefits in construction projects	Lack of research on Agile outside IT	Literature review, case studies	Agile can improve project flexibility, reduce delays, and enhance stakeholder satisfaction in construction
Agile in Construction Projects	Small sample size, participant bias, high cost	Investigate Agile’s challenges and potential in construction	Limited studies on Agile’s obstacles in construction	Qualitative interviews with 20 project managers	Agile offers benefits like improved flexibility and resource management but faces cultural resistance and high costs
Agile Frameworks in Construction Project Management	Limited access to non-English articles, incomplete data	Systematic review of Agile’s role in construction management	Few studies on Agile’s integration with Lean and BIM	Systematic review of 37 articles from 2006–2023	Agile frameworks boost productivity, cost control, and communication but face resistance to change in construction
A Framework for Utilization of Agile Management in Construction	Traditional methods lack flexibility and delay problem-solving.	Introduce Agile Project Management in construction.	Limited application of agile in construction projects.	Literature review, framework development, case studies.	Proposes a framework for agile use in construction to improve adaptability and efficiency.

## Material and method

**Fig. 4:**
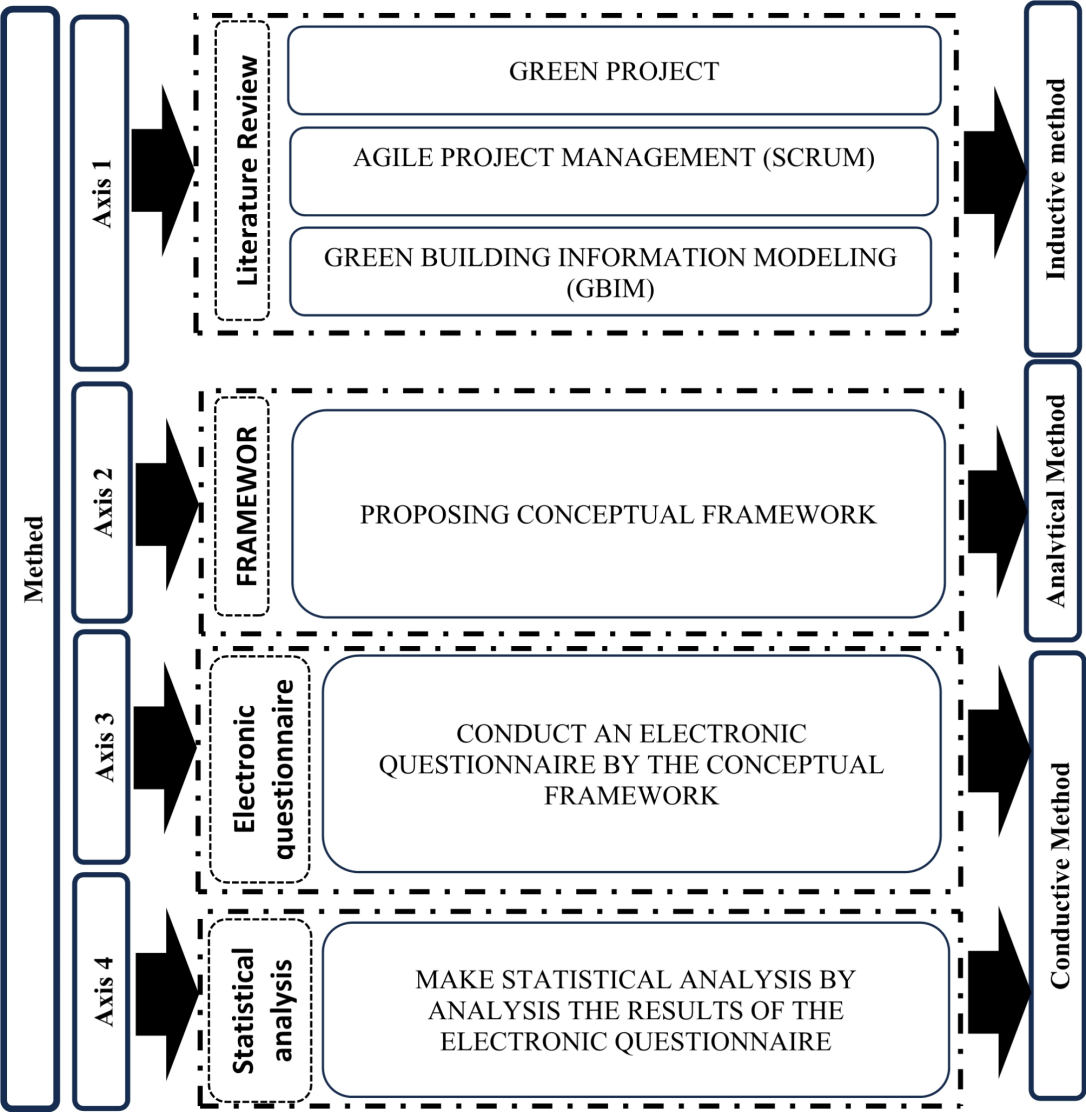
Illustration of the methodology consisting of 3 sections: inductive method, analytical method, and conductive method by author.

This research was conducted on four axes: according to Fig. 4.

**Fig. 5:**
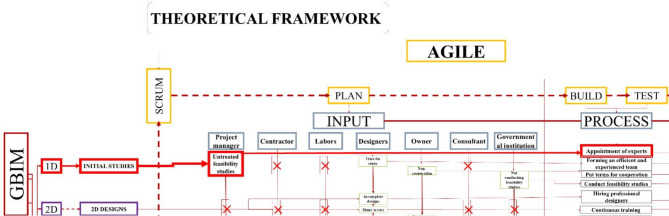
The first indicator and explains its work cycle, through the plan and then the process by author.

**Fig. 6:**
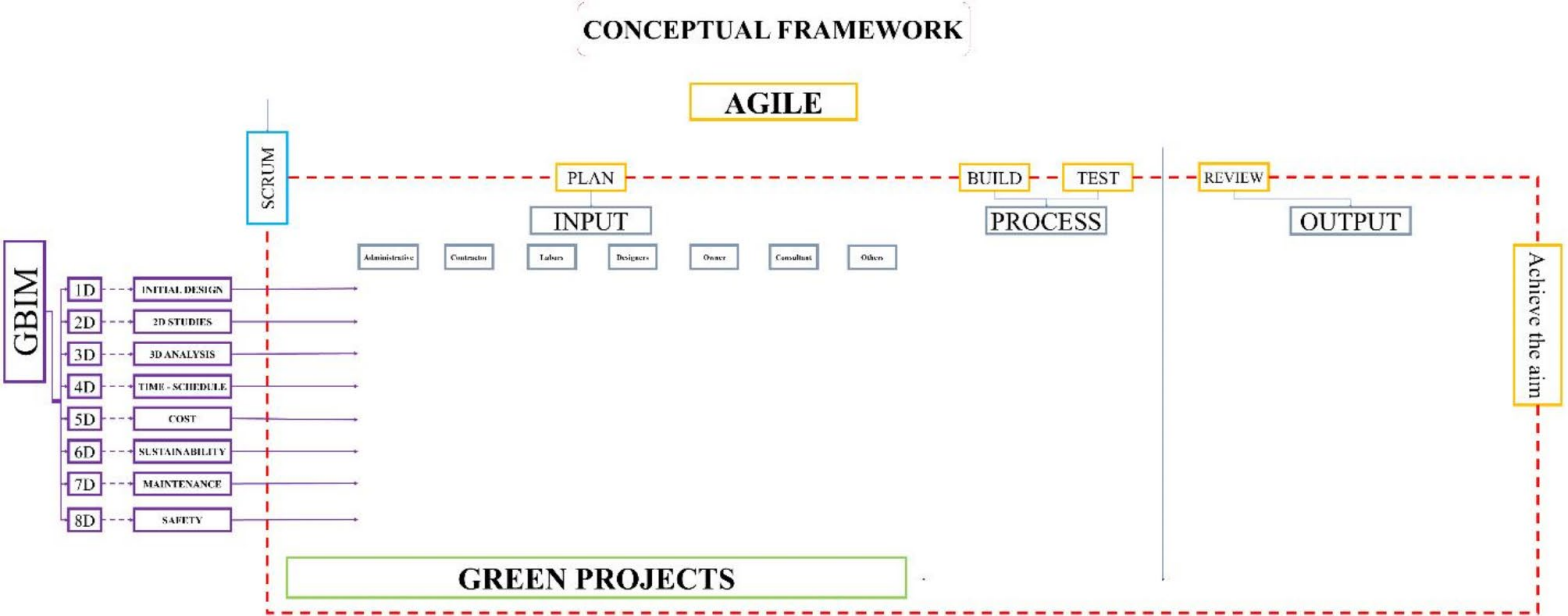
The Conceptual framework illustrates the relationship between the Agile processes represented in Scrum, GBIM, and green projects by dividing the work cycle into inputs, process, and outputs, and it can be repeated through Scrum by author.

- **The first axis**: Initially, a review of the literature was done on green projects because the primary goal of the research is to identify a framework and methodology that can anticipate and solve any issue that arises while also being updated on a regular basis. Because of its advantages in a smooth technique based on total error prevention, anticipation, and continuous improvement, the Agile methodology was chosen. Secondly, a review of the literature was done regarding the Agile methodology. The best approach from the AGILE methods was selected: Scrum. Because it isn’t as complex as other software methodologies, Scrum is the closest method that can be used for green projects and contracting projects in general. Lastly, because BIM technology is thought to be a bridge between green projects and the AGILE approach, it was selected as a tool to apply and implement the Scrum methodology on green projects. Consequently, a literature assessment was done specifically for the GBIM because it was determined to be more appropriate for green initiatives.

- **The second axis**: A Conceptual framework as in Fig. 6 is proposed based on the Scrum methodology, starting from planning through to review and achieving the final aim. This framework involves the development of a tool, the BIM tool, designed to organize and classify green projects into units ranging from 1D to 8D. The tool allows for input of all information related to green projects, categorized for all stakeholders, from government institutions to engineers and workers. This information includes potential problems and risks that both the stakeholders and the project may encounter. The inputs will drive the development process, with outputs derived according to the BIM tool’s principles, and key indicators will be extracted as a result of this process. The indicator was developed by connecting three elements, namely BIM from the dimensions-based classification and the Agile methodology, which is reflected in the workflow. Additionally, it incorporates inputs related to green projects, classified based on contributors to the process. For instance, in Fig. 5, indicator number (1) was generated by linking (1d), representing the initial studies, with the first input, the project manager, who is responsible for the feasibility studies conducted during the project’s preliminary phase.

- **The third axis**: The electronic questionnaire was designed to evaluate 30 indicators, as detailed in Table 4. These indicators were derived from the risks highlighted in the literature review, which identified a total of 30 risks gathered from an analysis of the challenges faced by green projects in various countries, as outlined in Table 5. The questionnaire consists of 30 primary questions, each corresponding to one of the indicators. The number 30 was selected based on the total outputs derived from all inputs, including all project-related data and information. This number may be adjusted in the future depending on the specific circumstances. These 30 indicators were used to create questions that evaluate their impact and possible application within the theoretical framework. The questionnaire began by collecting information such as name, email, specialization, and academic degree, along with a question identifying the key contributors in the design process. Following this, the 30 questions were categorized according to the project’s contributors, with all questions listed in appendix (1), as shown in supplementary Tables 1 and 2. Additionally, 140 experts in green technology, project management, and BIM technology, along with academics in these fields, were selected as participants, as indicated in Table 3. The participants were divided based on their representation in Egypt, calculated using Cochran’s sample size formula and the connectionist approach, as shown in Eq. (1)^49^:.

1 $$\text{n}=\frac{N{t}^{2}pq}{N{d}^{2}+{t}^{2}pq}\cdot^{49}$$

The variables used are as follows: n = sample size, N = population size, p = probability of success, t = confidence level value, q = probability of failure, and d = acceptable margin of error. We contacted accredited organizations and centers within the country to obtain approximate figures for the number of specialists. The data revealed that there are around 15,000 specialists in green projects, 4,000 specialists in BIM technology, and 300 specialists in Agile methodologies. Additionally, there are approximately 100 faculty members, including professors and assistant lecturers, bringing the total to 19,500 individuals. After applying these values to the equation, where *N* = 19,500, t = 1.95, *p*= 0.5, q = 0.5, and d = 0.07, the calculated result was 138, so 140 samples were selected for the questionnaire^49^.

$$\text{n}=\frac{19500{*1.95}^{2}*0.5*0.5}{19500{0.07}^{2}+{1.95}^{2}*0.5*0.5}=138=140\: \text{sample}$$

**Table 3:** Distribution of participants in the electronic questionnaire by author.

Participants	Number
Experts in green projects	90
Experts in BIM technology	25
Experts in AGILE management	15
Academic stuff (Doctors)	5
Academic stuff (Teaching assistant)	5
Total	140

**Table 4:** 30 indicators by author.

	Stakeholders	No.	Indicators
**A**	**Project manager**	1	(Experts role) feasibility studies/ green projects
		2	(Time) proactive system/green projects
		3	(Cost) AGILE methodology / green projects
		4	(Flexibility in change) AGILE methodology / green projects
		5	(Flexibility in change) AGILE methodology / life cycle buildings
		6	(Flexibility in change) AGILE methodology / safety and quality control
**B**	**Contractor**	1	(Time control process) Agile / project handover date
		2	(Competencies) AGILE / cost of waste
		3	(Environnemental context) Project implementation / quality
		4	(Continuous monitoring) AGILE approach / site management
**C**	**Labor**	1	(Continuous monitoring) AGILE approach / labor productivity
		2	(Reducing the errors) safety / site
**D**	**Designer**	1	(Time) specialized team / studies
		2	(Owner) professional team leader / innovative designs
		3	(Continuous improvement) design processes / reducing errors
		4	(Coordination) conflicts / drawings
		5	(Time management system) delays / drawings
		6	(Material) environmentally friendly / both thermal and acoustic comfort
		7	(Material) environmentally friendly / extending the life cycle of the building
**E**	**Owner**	1	(Quality of the project) cooperation / stakeholders
		2	(Reducing waste) parameters / continuous change
		3	(Owner) several suitable alternatives / preventing hesitations
		4	(Contract) financing conditions and strategies/organizing financing for the project
		5	(Green projects) high value / implement it in his project
		6	(Flexible programs) wasting / design stage
**F**	**Consultants**	1	(Competencies) pricing process / preventing errors
		2	(Competencies) supervision process / preventing errors
**G**	**Governmental ** **institution**	1	(Green projects) value / developing legislation
		2	(Green projects) value / apply it in their projects
		3	(Feasibility studies) great success / project

**Table 5:** Showing the creation of the 30 indicators from the 30 risks.

	Stakeholders	No.	Risks	Indicators
**A**	**Project manager**	1	Inaccurate studies	(Experts role) feasibility studies/ green projects
		2	Unpredictability	(Time) proactive system/green projects
		3	High cost of green projects	(Cost) AGILE methodology / green projects
		4	Lack of knowledge of green thought	(Flexibility in change) AGILE methodology / green projects
		5	Lack of interest in the life cycle of the building	(Flexibility in change) AGILE methodology / life cycle buildings
		6	Safety and accidents	(Flexibility in change) AGILE methodology / safety and quality control
**B**	**Contractor**	1	Delay in project delivery	(Time control process) Agile / project handover date
		2	Lots of waste during implementation	(Competencies) AGILE / cost of waste
		3	Lack of experience and quality	(Environnemental context) Project implementation / quality
		4	Poor site management	(Continuous monitoring) AGILE approach / site management
**C**	**Labor**	1	Labor productivity	(Continuous monitoring) AGILE approach / labor productivity
		2	Hazards facing workers on site	(Reducing the errors) safety / site
**D**	**Designer**	1	Poor team experience	(Time) specialized team / studies
		2	Poor in design	(Owner) professional team leader / innovative designs
		3	Errors in deign	(Continuous improvement) design processes / reducing errors
		4	Clashes in the design	(Coordination) conflicts / drawings
		5	Delay in project delivery	(Time management system) delays / drawings
		6	Lack of knowledge of materials related to thermal and acoustic comfort	(Material) environmentally friendly / both thermal and acoustic comfort
		7	Not knowing the materials that extend the life of the building	(Material) environmentally friendly / extending the life cycle of the building
**E**	**Owner**	1	Non-cooperation	(Quality of the project) cooperation / stakeholders
		2	Continuous change	(Reducing waste) parameters / continuous change
		3	Hesitation in making decisions	(Owner) several suitable alternatives / preventing hesitations
		4	Lack of a clear financing strategy	(Contract) financing conditions and strategies/organizing financing for the project
		5	Lack of knowledge of the value of green projects	(Green projects) high value / implement it in his project
		6	Failure to set flexible programs to reduce waste	(Flexible programs) wasting / design stage
**F**	**Consultants**	1	Errors in the pricing process	(Competencies) pricing process / preventing errors
		2	Error in the review process	(Competencies) supervision process / preventing errors
**G**	**Governmental ** **institution**	1	Change in regulations^6^	(Green projects) value / developing legislation
		2	Lack of awareness of the importance of green projects^8^	(Green projects) value / apply it in their projects
		3	Lack of interest in feasibility studies^15^	(Feasibility studies) great success / project

- **The fourth axis**: Statistical analysis was conducted to evaluate the relative significance of the electronic questionnaire results. The process started with calculating the mean (µ), standard deviation (σ), and coefficient of variance (CV) to gauge the homogeneity of the sample. Following this, the relative importance index (RII) was determined based on Likert scale classifications (k)^50–52^: “(EI) represents Extremely Important, (I) stands for Important, (A) for Average, (NI) for Not Important, and (ENI) for Extremely Not Important. Finally, the study assessed the level of importance, relative ranking, and percentage of global ranking for each of the 30 indicators using the following Eqs. (2, 3, 4).”^53–55^:

2 $$(\mu)=\text{n}1 +2\text{n}2 +3\text{n}3+4\text{n}4+ 5\text{n}5 / {\text{Total} \:\text{number} \:\text{of}\: \text{samples}}^{56}$$

3 $$(\text{CV})=(\alpha/\mu)* 100$$

Concerning the CV result, a value between 10 and 20 suggests that the sample was homogeneous and well-balanced. It’s important to note that a CV value^56^ below 10is regarded as an indication of an excellent sample, as outlined below^57^:

- C V < 10: Excellent sample.
- C V between 10 and 20: Very good.
- C V between 20 and 30: Acceptable.
- C V between 30 and 40: Low.
- CV > 40: Unacceptable.

4 $$(\text{RII})=\text{n}_{1}+\text{2n}_{2}+\text{3n}_{3}+\text{4n}_{4}+\text{5n5/5}(\text{n}_{1}+\text{n}_{2}+\text{n3}+\text{n4}+\text{n5})^{56}$$

- RII = 0.00-0.20: Importance Level (Low = L).
- RII = 0.21–0.40: Importance Level (Medium Low = ML).
- RII = 0.41–0.60: Importance Level (Medium = M).
- RII = 0.61–0.80: Importance Level (Medium High = MH).
- RII = 0.81-1.00: Importance Level (High = H)^58–60^.

The number of experts who assigned scores of ‘EI’ is (n5), ‘I’ is (n4), ‘A’ is (n3), ‘NI’ is (n2), and ‘ENI’ is (n). These experts were classified into four categories: those associated with BIM, represented by (B); those focused on agile methodologies, represented by (A); those involved in green architecture, represented by (G); and, lastly, academic experts, represented by (C). Based on the theoretical framework and the identified indicators, which are regarded as solutions to the challenges faced by green projects using BIM, these indicators were categorized into seven sections, as shown in Table 4. To gather expert opinions on the effectiveness of these indicators in addressing the issues of green projects, a baseline survey was conducted through an electronic questionnaire. The questionnaire consisted of 30 questions derived from the 30 indicators, and it was distributed to a sample of 70 experts. The complete results are presented in Table 7.

## Result

### Questionnaire analysis

The results are derived from an electronic questionnaire and expert evaluations addressing challenges in green projects. These findings are influenced by the integration of BIM technology and the Scrum framework within the Agile methodology. Statistical analysis was used to assess the electronic questionnaire, focusing on indicators related to stakeholders in green project challenges, and verified using the coefficient of variation (CV). The results indicated that group A was ranked as high (H), groups D and E as medium to high (M-H), groups B and F as medium (M), and group G as medium to low (M-L), as detailed in Tables 6 and 7.

Similarly, the overall indicators were ranked high (H) in the project manager category. Additionally, three indicators were ranked high (H) and one medium-high (M-H) under the contractor category. The indicators were also ranked high (H) for labor, designers, and consultants. For owners, five indicators were ranked high (H) and one medium-high (M-H). Lastly, the indicators for governmental institutions were ranked high (H). In conclusion, 28 indicators were ranked high (H) and two were ranked medium-high (M-H), as illustrated in Fig. 9.

### Validation

**Fig. 7:**
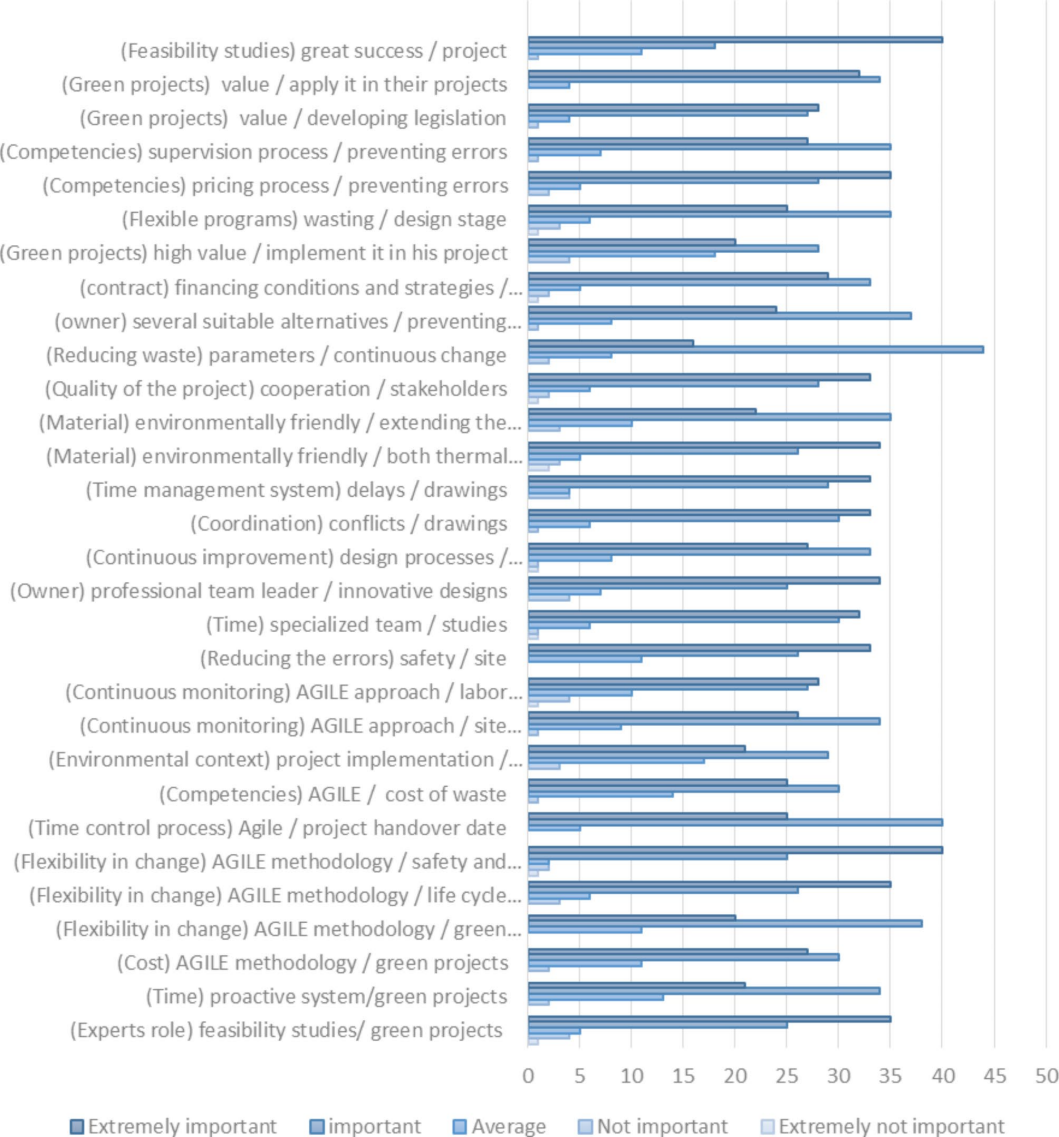
Experts’ overall evaluation for the indicators by author.

The CV result of **13.592** indicates a strong and balanced outcome, as shown in Table 7. Additionally, the results from the electronic questionnaire were illustrated with a chart highlighting the predominant trends in the responses. This visualization is crucial, as it emphasizes the two most significant and influential directions in the answers, as shown in Fig. 7.

### Result analysis

The findings are presented through tables and figures derived from the questionnaire data:

**First**,** in **Table 6:

**Table 6:** Indicators Model assessment by author.

	EI	I	A	NI	ENI		µ	(x̄-x)^2	α	cv		RII	RK
Group (A)	46	70	20	2	2	140	4.114	45.086	0.808	19.647	0.823	High	1
Group (B)	14	12	14	40	60	140	2.143	122.571	1.333	62.198	0.429	Medium	5
Group (C)	6	12	18	24	80	140	1.857	98.571	1.195	64.358	0.371	Medium Low	6
Group (D)	54	24	20	16	10	140	3.800	111.200	1.269	33.408	0.760	Medium High	2
Group (E)	14	32	60	20	10	140	3.171	73.943	1.035	32.641	0.634	Medium High	3
Group (F)	4	18	60	28	30	140	2.557	77.271	1.058	41.384	0.511	Medium	4
Group (G)	2	6	12	40	80	140	1.643	58.071	0.917	55.841	0.329	Medium Low	7
										**7.737**			

The indicators were categorized into seven groups based on the classification of project contributors. As a result, the calculated CV was **7.73**, which is considered an excellent outcome. A relative ranking was then assigned to each group, allowing the importance level of each group to be determined.

**Second**,** in** Table 7:

**Table 7:** The statistical analysis of the electronic questionnaire result by author.

		EI	I	A	NI	ENI		Mean	(x̄-x) ^2	α	CV	RII		RK
								µ					Importance level	
	**Project manager**													
A1		70	50	10	8	2	140	4.271	59.843	0.931	21.803	0.854	High	3
A2		42	68	26	4	0	140	4.057	41.771	0.778	19.178	0.811	High	6
A3		54	60	22	4	0	140	4.171	43.943	0.798	19.131	0.834	High	4
A4		40	76	22	2	0	140	4.100	34.300	0.705	17.196	0.820	High	5
A5		70	52	12	6	0	140	4.329	45.443	0.812	18.748	0.866	High	2
A6		80	50	4	4	2	140	4.443	45.271	0.810	18.232	0.889	High	1
	**Contractor**							0.000		0.000				
B1		50	80	10	0	0	140	4.286	24.286	0.593	13.843	0.857	High	1
B2		50	60	28	2	0	140	4.129	41.843	0.779	18.862	0.826	High	3
B3		42	58	34	6	0	140	3.971	49.943	0.851	21.422	0.794	Medium High	4
B4		52	68	18	2	0	140	4.214	35.786	0.720	17.089	0.843	High	2
	**Labors**							0.000		0.000				
C1		56	54	20	8	2	140	4.100	62.300	0.950	23.176	0.820	High	2
C2		66	52	22	0	0	140	4.314	37.086	0.733	16.993	0.863	High	1
	**Designers**							0.000		0.000				
D1		64	60	12	2	2	140	4.300	44.700	0.805	18.718	0.860	High	3
D2		68	50	14	8	0	140	4.271	51.843	0.867	20.293	0.854	High	4
D3		54	66	16	2	2	140	4.200	45.200	0.809	19.271	0.840	High	5
D4		66	60	12	2	0	140	4.357	34.071	0.703	16.128	0.871	High	2
D5		66	58	8	8	0	140	4.300	28.000	0.637	18.829	0.900	High	1
D6		68	52	10	6	4	140	4.243	28.000	0.637	18.829	0.900	High	1
D7		44	70	20	6	0	140	4.086	28.000	0.637	18.829	0.900	High	1
	**Owners**							0.000		0.000				
E1		66	56	12	4	2	140	4.286	50.286	0.854	19.919	0.857	High	1
E2		32	88	16	4	0	140	4.057	31.771	0.679	16.725	0.811	High	5
E3		48	74	16	2	0	140	4.200	33.200	0.694	16.516	0.840	High	3
E4		58	66	10	4	2	140	4.243	46.871	0.824	19.425	0.849	High	2
E5		40	56	36	8	0	140	3.914	53.486	0.880	22.493	0.783	Medium High	6
E6		50	70	12	6	2	140	4.143	50.571	0.856	20.665	0.829	High	4
	**Consultants**							0.000		0.000				
F1		70	56	10	4	0	140	4.371	38.343	0.745	17.053	0.874	High	1
F2		54	70	14	2	0	140	4.257	33.371	0.695	16.336	0.851	High	2
	**Governmental institution**							0.000		0.000				
G1		76	54	8	2	0	140	4.457	31.371	0.674	15.128	0.891	High	2
G2		64	68	8	0	0	140	4.400	11.500	0.408	11.432	0.950	High	1
G3		80	36	22	2	0	140	4.386	11.500	0.408	11.432	0.950	High	1
											**13.592**			

Secondly, in Table 7, all the indicators were listed, their importance was assessed, and a relative ranking was assigned to each group of related indicators.

**Third**, Fig. 7:

Presents a chart displaying the importance levels for all indicators, accompanied by a key for each corresponding importance rate.

**Fourth**, Fig. 8:

**Fig. 8:**
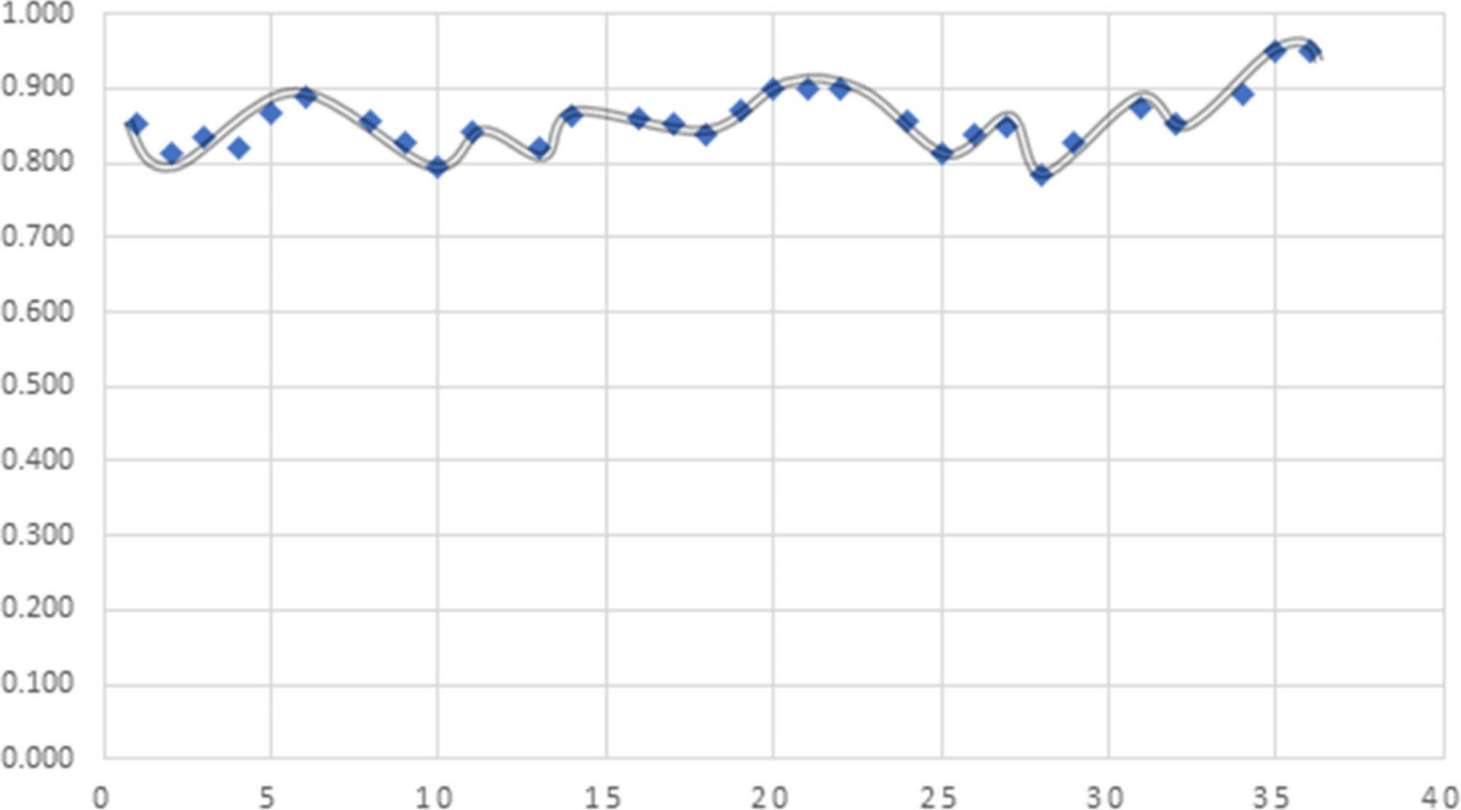
RII results by author.

The graph illustrates the importance level, indicating that the RII values for all indicators range from 0.8 to 0.98. This suggests that the indicators fall between medium high importance and high importance.

**Fifth**, Fig. 9:

**Fig. 9:**
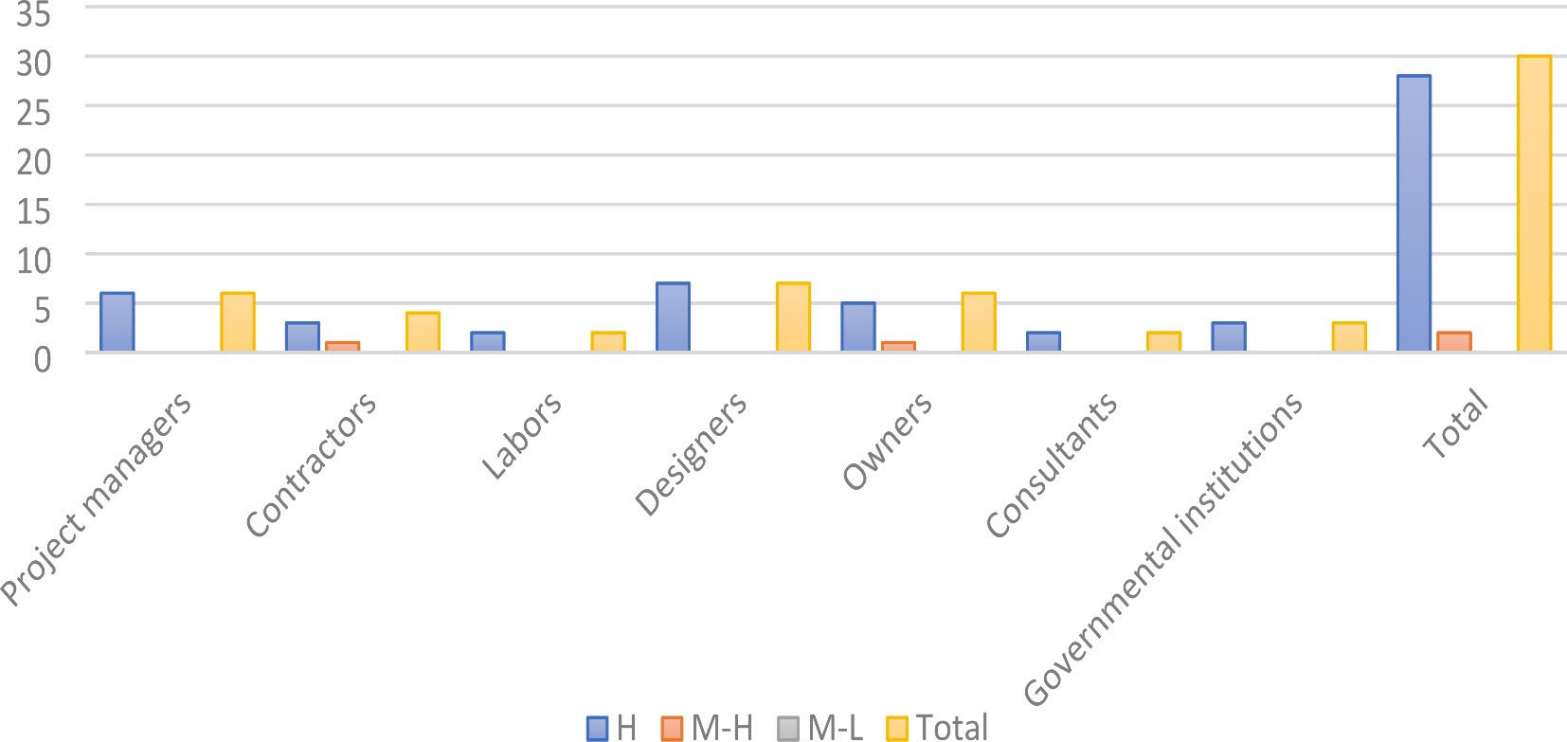
The importance level for the risk factors by author.

The graph illustrates the significance of various groups of indicators associated with contributors to green projects, highlighting which group has the greatest influence on these initiatives.

Finally, the results of the study highlight the effective integration of Agile methodologies, particularly Scrum, and Green Building Information Modeling (GBIM) in managing green projects. The high ranking of indicators related to time control, cost efficiency, and flexibility underscores the importance of these tools in addressing the challenges of green projects, such as delays, cost overruns, and stakeholder coordination. The literature emphasizes similar findings, showing that Agile enhances project flexibility and responsiveness, while GBIM improves data management and decision-making. Despite these strengths, challenges such as knowledge gaps among project participants, especially contractors and laborers, remain, which could be addressed through targeted training and better use of GBIM to provide real-time project data.

For practice, the study suggests that improving stakeholder engagement, particularly among governmental institutions, owners, and consultants, is critical to the success of green projects. The integration of Agile and GBIM provides opportunities for continuous improvement, reducing project risks and improving sustainability outcomes. For future research, more exploration is needed on the scalability of Agile methodologies in large-scale green projects and the adoption of GBIM in developing countries. Additionally, fostering cross-disciplinary collaboration among stakeholders could enhance the effectiveness of these integrated approaches in managing green projects.

## Analysis and discussion

According to the relative ranking, each indicator has ranking according to its own section, where the ranking is done according to the value of the RII, and thus the ranking is made starting from the first section, which is the project manager, which contains 6 indicators, as the flexibility in change is specific to the Agile methodology and is related to safety and quality control is ranked 1, and those related to the building’s life cycle are ranked 2, but those related to green projects are ranked 5, and also the feasibility studies related to green projects are ranked 3, and the indicator related to the cost of green projects is ranked 4, and finally the indicator related to time is ranked 6. As for the second section on the contractor, which contains 4 indicators, the indicator on project delivery time was ranked 1, while the part on continuous monitoring was ranked 2, and the two indicators on competencies and environmental quality were ranked 3 and 4. In the third section on labor, which contains only two indicators, the indicator on site safety is ranked 1, while the indicator on labor productivity is ranked 2. And the fourth section on designers, which contains 7 indicators, the three indicators on the time management system and environmentally friendly materials related to thermal and acoustic comfort and the life cycle building are ranked 1, the indicator for coordination is ranked 2, the part related to the time of submitting studies is ranked 3, the indicator for innovative designs is ranked 4, and finally the part related to continuous improvement is ranked 5. The section on owners, which contains 6 indicators, where the indicator on cooperation between the owner and work personnel is ranked 1, the indicator related to organizing financing is ranked 2, and also the indicator related to preventing hesitation in choosing is ranked 3, while the indicator related to flexible programs is ranked 4, the indicator related to continuous change is ranked 5, and finally the part related to knowing the high value of green projects is ranked 6.

Finally, the two sections are for consultants and government institutions. The section for consultants contains only two indicators. The indicator for the pricing process is ranked 1, while the indicator for the supervision process is ranked 2. The section for government institutions contains three indicators, where the two indicators for feasibility studies and knowing the value of green projects are ranked 1, while the indicator for developing laws and legislation is ranked 2.

The theoretical framework developed in this study offers significant practical benefits for project managers overseeing green projects. By integrating Agile methodologies, specifically Scrum, with GBIM (Green Building Information Modeling), project managers are equipped to better manage risks such as cost overruns, delays, and stakeholder coordination, which are common in sustainable construction. The iterative nature of Scrum allows for real-time adjustments and flexibility, which is essential in responding to the dynamic environmental and technical challenges of green projects. Additionally, the framework’s emphasis on continuous monitoring and feedback ensures that sustainability goals are maintained throughout the project lifecycle. For instance, the real-time data provided by GBIM enables project managers to optimize resource usage, improve energy efficiency, and reduce waste. This application not only enhances the project’s environmental performance but also improves overall project efficiency. The ability to anticipate and resolve issues early through Agile’s structured sprints aligns with the principles of sustainable project management, ensuring that green initiatives can be delivered on time and within budget, while adhering to environmental standards. Consequently, this framework enhances the project manager’s capability to deliver high-quality, sustainable outcomes, reinforcing the practical utility of the research findings in real-world green projects.

## Theoretical framework

**Fig. 10:**
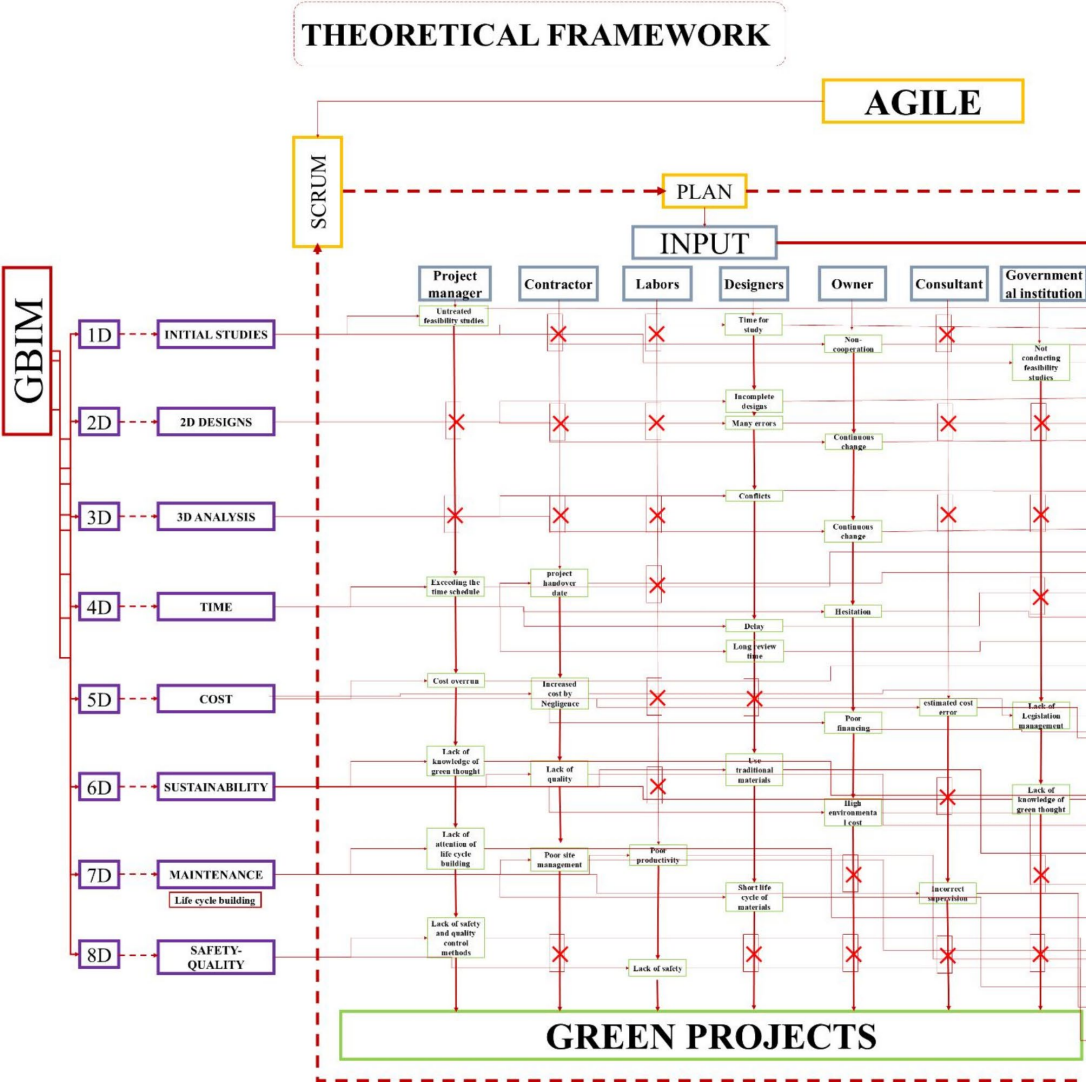
Theoretical framework, showing the development of indicators through the process of linking between Agile, GBIM and green projects in the form of inputs, process and output’s part 1 by author.

**Fig. 11:**
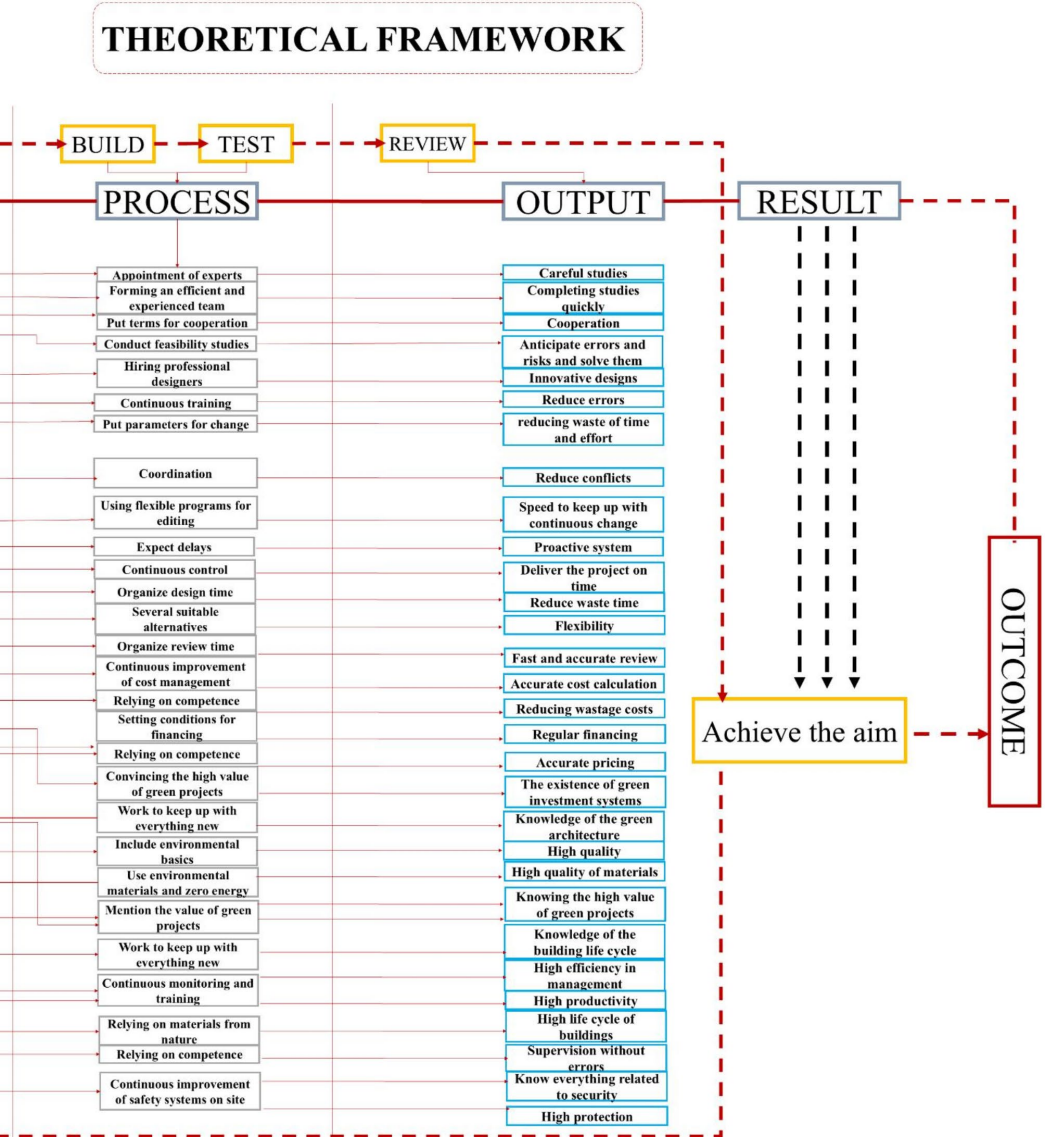
Theoretical framework, showing the development of indicators through the process of linking between Agile, GBIM and green projects in the form of inputs, process and outputs part 2 by author.

Using an electronic questionnaire and statistical analysis of the data, a theoretical framework was established. This framework offers insights and estimates that are applicable to green projects. By adhering to this framework, it becomes possible to foresee and address many of the challenges that green projects might encounter, as illustrated in Figs. 10 and 11.

## Recommendations

This research proposes several recommendations: First, the theoretical framework can be transformed into a practical one, incorporating indicators that assess all aspects of green projects. After evaluation, proposals and solutions can be offered to address the challenges and risks faced by the project. Second, in relation to BIM technology and the Agile methodology, the framework can be adjusted to keep up with the rapid advancements in BIM and updates in Agile practices. Third, for future research building on our work, it is possible to integrate BIM technology with artificial intelligence, which is advancing rapidly and having a significant impact on green projects. Additionally, BIM technology could be linked with the Seven Sigma methodology to explore its effects on green projects. Furthermore, future research should explore the application of Agile methodologies and GBIM across different scales of green projects, such as large-scale infrastructure developments and small-scale sustainable buildings. For large-scale projects, research could investigate how Agile frameworks, like Scrum, can be scaled up to enhance coordination among diverse stakeholders and manage the complexity of environmental standards. Conversely, small-scale projects may benefit from Agile’s flexibility in rapid iterations and its ability to adapt to changing sustainability goals. Studies could also evaluate the long-term impacts of GBIM in maintaining energy efficiency and reducing lifecycle costs in different project sizes, providing clearer insights into optimizing these tools for varied project environments.

## Conclusions

Given their environmental, economic, and social significance, green projects are crucial endeavors that warrant global attention. Expanding their implementation worldwide is essential. To achieve this, it is critical to address and mitigate the risks and challenges associated with green projects both before and during their implementation. This study suggests utilizing the Agile methodology, which offers significant advantages in reducing errors and promoting continuous improvement. The integration of Agile with BIM (Building Information Modeling) technology is proposed to tackle these challenges. An electronic questionnaire and expert evaluations were conducted to refine the conceptual framework, which merges Agile methodology with BIM technology to overcome obstacles in green project implementation.

The results show that most indicators were rated as either of high or medium importance, particularly those involving key stakeholders like project managers, consultants, designers, and government institutions. The indicator related to contractors was rated as medium importance, suggesting that the selected indicators carry substantial weight and maintain a stable position within the theoretical framework, given their significant influence on the ongoing improvement of green projects. Indicators associated with government institutions, project managers, consultants, and other decision-makers are especially crucial, as they impact green projects and help mitigate potential issues. After the analysis, these indicators were ranked, with the most important indicators identified first, and their impact on the outcomes was assessed.

The study found that the Agile methodology is effective in resolving issues by facilitating coordination to prevent conflicts between departments, ensuring continuous improvement, and maintaining ongoing monitoring to anticipate previously encountered risks. Moreover, Agile promotes enhanced collaboration among all project disciplines, including the owner, and ensures the careful selection and proper accreditation of employees. It is also proficient at integrating expertise and developing ongoing training plans for project implementation. A key principle of Agile is its flexibility in adapting to changes and staying current with innovations. This includes using BIM technology to classify problems by dimensions and ensure alignment with the technological approach to project implementation. BIM technology was instrumental in developing viable solutions.

The research recommends transforming the theoretical framework into a practical one ready for application in green projects by converting indicators into evaluation elements for these projects. Additionally, it suggests further development of the framework to keep pace with advancements in BIM technology.

## Electronic supplementary material

Below is the link to the electronic supplementary material.

Supplementary Material 1

## Data Availability

The datasets used and/or analyzed during the current study are available from the corresponding author upon reasonable request.

## References

[1] Wasiuzzaman, S., Hj Pungut, N. N. & Md Don, M. K. S. Crowdfunding green projects in Brunei: awareness and investing preferences. *Manag Environ. Qual. Int. J.* **32** (6), 1383–1400. 10.1108/MEQ-03-2021-0046 (2021).

[2] Hwang, B. G. & Leong, L. P. Comparison of schedule delay and causal factors between traditional and green construction projects. *Technol. Econ. Dev. Econ.* **19** (2), 310–330. 10.3846/20294913.2013.798596 (2013).

[3] Ahmad, T., Aibinu, A. A. & Stephan, A. Managing green building development – A review of current state of research and future directions. *Build. Environ.* **155** (January), 83–104. 10.1016/j.buildenv.2019.03.034 (2019).

[4] Reyes Maturano, I. Social Dispossession, the real ‘Benefit’ of Green projects in Yucatan. *Dev*. **65** (1), 63–70. 10.1057/s41301-021-00298-w (2022).

[5] Smirnova, O. & Vilenskii, M. P rinciples of Green Architecture for the historical part of saint-petersburg. *Architectura*. **10** (2), 103–112 (2019).

[6] Zhao, X., Hwang, B. G. & Gao, Y. A fuzzy synthetic evaluation approach for risk assessment: a case of Singapore’s green projects. *J. Clean. Prod.* **115** (January 2005), 203–213. 10.1016/j.jclepro.2015.11.042 (2016).

[7] Green, T. O. A., Muhammedjanovna, D. G. & UZBEKISTAN’S TRANSITION TO A GREEN ECONOMY PROBLEMS AND SOLUTIONS Davlatova. *J. Sci. Res. Mod. VIEWS Innov.* **1** (1), 34–39 (2024).

[8] Huang, L., Bai, B. & Ji, J. The present situation, problems and countermeasures of green investment in China. *IOP Conf. Ser. Earth Environ. Sci.* **427** (1). 10.1088/1755-1315/427/1/012014 (2020).

[9] Hazzan, O. & Dubinsky, Y. The Agile Manifesto. *SpringerBriefs Comput. Sci.* **0** (9783319101569), 9–14. 10.1007/978-3-319-10157-6_3 (2014).

[10] Oyedepo, S. O. On energy for sustainable development in Nigeria. *Renew. Sustain. Energy Rev.* **16** (5), 2583–2598. 10.1016/j.rser.2012.02.010 (2012).

[11] Cesarotti, V., Gubinelli, S. & Introna, V. The evolution of Project Management (PM): how agile, lean and six Sigma are changing PM. *J. Mod. Proj Manag*. **7** (3), 162–189. 10.19255/JMPM02107 (2019).

[12] Rahardjati, R., Khamidi, M. F. & Idrus, A. The level of importance of criteria and sub criteria in Green Building Index Malaysia. *Civ. Eng.* ;(May):1–6. (2009).

[13] Vierra, S. Green Building standards and Certification Systems|WBDG - Whole Building Design Guide. *Natl. Inst. Build. Sci. Published Online* **2018**:1–51. https://www.wbdg.org/resources/green-building-standards-and-certification-systems

[14] Maltzman, R. & Shirley, D. Green project management. *Green. Proj Manag Published Online* **2010**:1–280. 10.1201/EBK1439830017

[15] Nguyen, H. D. & Macchion, L. A comprehensive risk assessment model based on a fuzzy synthetic evaluation approach for green building projects: the case of Vietnam. *Eng. Constr. Archit. Manag*. **30** (7), 2837–2861. 10.1108/ECAM-09-2021-0824 (2023).

[16] Hwang, B. G. & Tan, J. S. Green building project management: obstacles and solutions for sustainable development. *Sustain. Dev.* **20** (5), 335–349. 10.1002/sd.492 (2012).

[17] Ahn, Y. H., Pearce, A. R., Wang, Y. & Wang, G. Drivers and barriers of sustainable design and construction: the perception of green building experience. *Int. J. Sustain. Build. Technol. Urban Dev.* **4** (1), 35–45. 10.1080/2093761X.2012.759887 (2013).

[18] Zainul Abidin, N. & Amir Shariffuddin, N. A. Engaging consultants in green projects: exploring the practice in Malaysia. *Smart Sustain. Built Environ.* **8** (1), 80–94. 10.1108/SASBE-06-2018-0033 (2019).

[19] Improta, G. et al. Agile six sigma in healthcare: case study at santobono pediatric hospital. *Int. J. Environ. Res. Public. Health*. **17** (3). 10.3390/ijerph17031052 (2020). 10.3390/ijerph17031052 PMC7037742 32046052

[20] Magistretti, S. & Trabucchi, D. *Agile-as-a-Tool and Agile-as-a-Culture: A Comprehensive Review of Agile Approaches Adopting Contingency and Configuration Theories* (Springer, 2024). 10.1007/s11846-024-00745-1

[21] Mohammed, K. N. & Karri, S. C. An analytical approach in usage of agile methodologies in construction industries - A case study. *Mater Today Proc*. ;33(xxxx):475–479. doi: (2020). 10.1016/j.matpr.2020.05.045

[22] Wisdom Ebirim, D. J. P., Montero, E. C. & Ani Nwakamma Ninduwezuor-Ehiobu, Favour Oluwadamilare Usman, Kehinde Andrew Olu-lawal. The role of Agile Project Management in Driving Innovation in energy-efficient Hvac solutions. *Eng. Sci. Technol. J.* **5** (3), 662–673. 10.51594/estj.v5i3.864 (2024).

[23] Pimonova, S. Agile methodology in education of IT students, application of. *Encycl Educ Inf Technol*. *Published Online* **2020**:1–10. 10.1007/978-3-319-60013-0_214-1

[24] Streule, T., Miserini, N., Bartlomé, O., Klippel, M. & De Soto, B. G. Implementation of Scrum in the Construction Industry. *Procedia Eng.* **164** (June), 269–276. 10.1016/j.proeng.2016.11.619 (2016).

[25] Srivastava, A. & Automation, I. C. C. C. A. Proceeding - IEEE International Conference on Computing, Communication and *Proceeding - IEEE Int Conf Comput Commun Autom ICCCA 2016*. Published online 2017:864–869. (2016).

[26] Mayo-Alvarez, L. et al. Innovation by integration of Drum-buffer-rope (DBR) method with Scrum-Kanban and use of Monte Carlo simulation for maximizing throughput in agile project management. *J. Open. Innov. Technol. Mark. Complex.* **10** (1). 10.1016/j.joitmc.2024.100228 (2024).

[27] Robert, B. & Brown, E. B. *Agile Project Management with Scrum*.; (2004).

[28] Pries, K. H. & Quigley, J. M. *Scrum Project Management*.; (2010).

[29] Vilca, Y. H. & León, J. B. Agile frameworks in Construction Project Management: a systematic review. *Proc. World Congr New. Technol. Published Online* **2024**:1–8. 10.11159/icceia24.114

[30] Mohamed, B. & Moselhi, O. A framework for utilization of agile management in construction management. *Proceedings, Annu Conf - Can Soc Civ Eng*. ;2019-June:1–10. (2019).

[31] Badran, S. S. & Abdallah, A. B. Lean vs agile project management in construction: impacts on project performance outcomes. *Eng. Constr. Archit. Manag* Published online 2024. 10.1108/ECAM-05-2023-0470

[32] Jurković, M. et al. doo PROJECT CONSULTING, obrt za poslovno savjetovanje, vl AGILE MANAGEMENT IN THE CONSTRUCTION SECTOR. Published online 2024:1–7.

[33] Kineber, A. F., Oke, A. E., Zamil, A. & Alhusban, M. Agile project management for sustainable residential construction: A study of critical success factors. ;(October):1–13. doi: (2024). 10.3389/fbuil.2024.1442184

[34] Kumar Jha, S. Annals of the Bhandarkar Oriental Research Institute. *UGC Care Gr 1 J.* **CIV** (7), 111 (2023).

[35] Jin, C. Agile in Construction projects. *Published Online* :18. (2017). http://digitalcommons.harrisburgu.edu/pmgt_dandt/26

[36] Jethva, S. S. & Skibniewski, M. J. Agile project management for design-build construction projects: a case study. *Int. J. Appl. Sci. Eng.* **19** (1), 1–11. 10.6703/IJASE.202203_19(1).001 (2022).

[37] Systems, R., Systems, C. & GREEN BIM – It ’ s Various Aspects and Future Potential for Construction of Green Building Projects.

[38] Al-Ashmori, Y. Y. et al. BIM benefits and its influence on the BIM implementation in Malaysia. *Ain Shams Eng. J.* **11** (4), 1013–1019. 10.1016/j.asej.2020.02.002 (2020).

[39] *Eur J …*. 2023;1(2):9–13. http://e-science.net/index.php/EJCBLT/article/view/87.

[40] Barnes, P. BIM in Principle and in practice. *BIM Princ Pract. Published Online*. 10.1680/bimpp.63693 (2019).

[41] Yang, M., Chen, S. & Xu, J. Information flow molding technology of GBIM. *Adv. Mater. Res.* **594–597**, 2886–2892. 10.4028/www.scientific.net/AMR.594-597.2886 (2012).

[42] Luo, X. S., Xu, M. & Gan, C. Research on collaborative design system of green building information model. *Adv. Mater. Res.* **1042**, 272–276. 10.4028/www.scientific.net/AMR.1042.272 (2014).

[43] Piaseckienė, G. Dimensions of Bim in Literature: review and analysis. *Moksl - Liet Ateitis*. **14** (0), 1–11. 10.3846/mla.2022.16071 (2022).

[44] Habib, H. M. & Kadhim, R. E. Employ 6D-BIM model features for buildings sustainability Assessment. *IOP Conf. Ser. Mater. Sci. Eng.* **901** (1). 10.1088/1757-899X/901/1/012021 (2020).

[45] Farmers, T. H. E., Helper, L. S., Ustainable, P., Arasite, M. & Anagement *World Constr Conf 2012 – Glob Challenges Constr Ind 28–30 June 2012, Colombo, Sri Lanka*. ;(June):10–11. (2007).

[46] Shang, J. Research on the development status, problems, and optimization path of Green Finance. *Commercial Banks*. **7** (April), 157–160. 10.55014/pij.v7i2.589 (2024).

[47] Control, P. P., Performance, P. M. & Contexts, D. Project Portfolio Control and Portfolio. *Proj Manag J.* **39** (4), 28–42. 10.1002/pmj (2008).

[48] Qian, Q. K., Chan, E. H. W. & Khalid, A. G. Challenges in delivering green building projects: unearthing the transaction costs (TCs). *Sustain*. **7** (4), 3615–3636. 10.3390/su7043615 (2015).

[49] Balali, A. & Valipour, A. Identification and selection of building façade’s smart materials according to sustainable development goals. *Sustain. Mater. Technol.* **26**, e00213. 10.1016/j.susmat.2020.e00213 (2020).

[50] Mediastika, C. & Lie, K. Occupants’ perception on Green-rated Office Building in Surabaya, Indonesia. *Procedia Eng.* **118**, 546–553. 10.1016/j.proeng.2015.08.479 (2015).

[51] Beyaz, Ç. & Asilsoy, B. Knowledge of green buildings and environmental worldview among interior design students. *Int. J. Adv. Appl. Sci.* **6** (1), 29–36. 10.21833/ijaas.2019.01.004 (2019).

[52] Liu, K. S., Liao, Y. T. & Hsueh, S. L. Implementing smart green building architecture to residential project based on Kaohsiung, Taiwan. *Appl. Ecol. Environ. Res.* **15** (2), 159–171. 10.15666/aeer/1502_159171 (2017).

[53] Joshi, A., Kale, S., Chandel, S. & Pal, D. Likert Scale: explored and explained. *Br. J. Appl. Sci. Technol.* **7** (4), 396–403. 10.9734/bjast/2015/14975 (2015).

[54] Selim, A. M. & Saeed, D. M. Infrastructure projects for green cities between implementation challenges and efficiency indicators. *Civ. Eng. Archit.* **9** (2), 347–356. 10.13189/cea.2021.090208 (2021).

[55] Selim, A. M. A new era for public–private partnership (PPPs) in Egypt’s urban water supply projects: risk assessment and operating model. *HBRC J.* **18** (1), 157–182. 10.1080/16874048.2022.2126209 (2022).

[56] Hatkar, K. & Hedaoo, N. Delay analysis by using relative importance index method in infrastructure projects. *Int. J. Civ. Eng. Concr Strutures*, **1**(*3*), 3. (2016).

[57] Zaki, S. Assessment of Sustainable Development Benchmarks for Remedial Slums’ projects Applied on Re-housing projects. *JES J. Eng. Sci.* **0** (0), 0–0. 10.21608/jesaun.2023.178233.1187 (2023).

[58] Engineering Science and Military Technologies Architecture and structure considerations for the high-risk buildings : Smart architectural model for safe security surveillance point ... Architecture and structure considerations for the high-risk buildings . 2022;(March 2021). doi:10.21608/JMTC.2021.80774.1188

[59] Saeed, D. M., Elkhatib, W. F. & Selim, A. M. Architecturally safe and healthy classrooms: eco-medical concept to achieve sustainability in light of COVID-19 global pandemic. *J. Asian Archit. Build. Eng.* **21** (6), 2172–2187. 10.1080/13467581.2021.1972811 (2022).

[60] Holt, G. D. Asking questions, analysing answers: relative importance revisited. *Constr. Innov.* **14** (1), 2–16. 10.1108/CI-06-2012-0035 (2013).

