# Supplementary material for: Developing a theoretical framework for enhancing green project approaches via Agile methodology
Source: Sci Rep. 2024 Nov 13;14:27786. doi: 10.1038/s41598-024-78613-x (PMC11561110; doi:10.1038/s41598-024-78613-x)
Supplement: Supplementary file 1 — Supplementary Material 1 [file 41598_2024_78613_MOESM1_ESM.docx]

**Appendix (1) for: Developing a Theoretical Framework for Enhancing Green Project Approaches via Agile Methodology**

**(Utilizing BIM as a Tool)**

Ahmed. Ali¹*, Hesham. Sameh²

1. Faculty of Engineering, Cairo University. ([ahmed.ibrahim201117@yahoo.com](mailto:ahmed.ibrahim201117@yahoo.com))

(PhD Candidate in Cairo university)

2. Faculty of Engineering, Cairo University

(Professor of architecture in Cairo university)

**Electronic questionnaire questions shown in supplementary tables 1 and 2**

| Email address |  | |
| --- | --- | --- |
| Name |  | |
| Field of specialization | Architects | () |
|  | Project manager | () |
|  | Environmental expert | () |
|  | Management expert | () |
|  | Government officials | () |
|  | Academic | () |
| Years of experience | Less than 5 | () |
|  | 5-10 | () |
|  | Greater than 10 | () |
| Important in the design process | The Project manager | () |
|  | The Contractor | () |
|  | The Labors | () |
|  | The Designers | () |
|  | The Owners | () |
|  | The Consultants | () |
|  | The Governmental institution | () |

**Supplementary table 1.** General information by author

| **INDICATORS** | | **EI** | **I** | **A** | **NI** | **ENI** |
| --- | --- | --- | --- | --- | --- | --- |
| **Project manager** | Is the preparation of feasibility studies by relevant experts effective for green projects? | () | () | () | () | () |
|  | Is a proactive system essential in ensuring that the green project is implemented on time? | () | () | () | () | () |
|  | Is the continuous improvement system of the AGILE methodology effective in ensuring that costs are not exceeded in green projects? | () | () | () | () | () |
|  | Is flexibility in change, knowledge and adaptation to everything new related to the AGILE methodology regarding to promote green projects by project managers? | () |  | () | () | () |
|  | Is flexibility in change, knowledge and adaptation to everything new related to the AGILE methodology fundamental in the knowledge of A: The life cycle buildings by project managers? | () | () | () | () | () |
|  | Is flexibility in change, knowledge and adaptation to everything new related to the AGILE methodology fundamental in the knowledge of B: The safety and quality control by project managers? | () | () | () | () | () |
| **Contractor** | Is continuous time control process related to agile effective in controlling project handover date? | ()() | () | () | () | () |
|  | Is relying on competencies and also improving their experience according to the basics of agile effective in reducing the cost of waste as a result of negligence? | () | () | () | () | () |
|  | Is including environmental context in the project implementation methods essential in achieving the highest quality by the contractor? | () | () | () | () | () |
|  | Is continuous monitoring and training regarding the Agile approach mandatory in improving site management? | () | () | () | () | () |
| **Labors** | Is continuous monitoring and training regarding the agile approach essential in improving labor productivity? | () | () | () |  | () |
|  | Is the approach based on reducing the errors to the lowest percentages that may be non-existent useful and effective in achieving the highest rates of safety on the site? | () | () | () | () | () |
| **Designers** | Is attention to preparing a specialized team with competence and experience consider significant in completing studies on time? | () | () | () | () | () |
|  | Is hiring professional team leader designer’s mandatory in producing complete, innovative designs that suit the owner? | () | () | () | () | () |
|  | Is continuous improvement in design processes capable of reducing errors? | () | () | () | () | () |
|  | Is coordination between all disciplines effective in preventing conflicts in drawings? | () | () | () | () | () |
|  | Is the work of a time management system effective in preventing delays in delivering drawings? | () | () | () | () | () |
|  | Is the use of environmentally friendly and sustainable materials essential in improving construction quality and achieving both thermal and acoustic comfort for users? | () | () | () | () | () |
|  | Is relying on environmentally friendly materials essential in extending the life cycle of the building? | () | () | () | () | () |
| **Owners** | Is cooperation with the stakeholders essential in achieving the highest possible quality of the project and achieving all his desires? | () | () | () | () | () |
|  | Is setting parameters for continuous change significant in reducing waste of time and effort at work? | () | () | () | () | () |
|  | Is presenting several suitable alternatives to the owner effective in preventing hesitation in choosing the ideal alternative? | () | () | () | () | () |
|  | Is setting financing conditions and strategies for financing in the contract mandatory in organizing financing for the project by the owner? | () | () | () | () | () |
|  | Is presenting the high value of green projects and the value of sustainability to the owner essential in his desire to implement them in his project? | () | () | () | () | () |
|  | Is using flexible programs for rapid modification effective in keeping up with all the owner’s ongoing modifications without wasting time and effort in the design stage? | () | () | () | () | () |
| **Consultants** | Is relying on the competencies and experiences of consultants effective in: A: Controlling the pricing process in tender phase and preventing errors in it? | () | () | () | () | () |
|  | Is relying on the competencies and experiences of consultants effective in: B: Controlling the supervision process and preventing errors in it? | () | () | () | () | () |
| **Governmental institution** | Is convincing government institutions of the high value of green projects fundamental in developing the necessary laws and legislation to expand their application? | () | () | () | () | () |
|  | Is presenting the high value of green projects and the value of sustainability to government institutions essential in convincing them to implement them in their projects? | () | () | () | () | () |
|  | Is the phase of preparing feasibility studies before starting the project effective in achieving great success for this project? | () | () | () | () | () |

**Supplementary table 2.** Electronic questionnaire questions by author
